# Changing motor perception by sensorimotor conflicts and body ownership

**DOI:** 10.1038/srep25847

**Published:** 2016-05-26

**Authors:** R. Salomon, N. B. Fernandez, M. van Elk, N. Vachicouras, F. Sabatier, A. Tychinskaya, J. Llobera, O. Blanke

**Affiliations:** 1 Center for Neuroprosthetics, École Polytechnique Fédérale de Lausanne, Switzerland; 2 Laboratory of Cognitive Neuroscience, Brain Mind Institute, École Polytechnique Fédérale de Lausanne, Switzerland; 3 Department of Psychology, University of Amsterdam, Netherlands; 4 Department of Computer Science, École Polytechnique Fédérale de Lausanne, Switzerland; 5 Department of Neurology, University Hospital Geneva, Switzerland

## Abstract

Experimentally induced sensorimotor conflicts can result in a loss of the feeling of control over a movement (sense of agency). These findings are typically interpreted in terms of a forward model in which the predicted sensory consequences of the movement are compared with the observed sensory consequences. In the present study we investigated whether a mismatch between movements and their observed sensory consequences does not only result in a reduced feeling of agency, but may affect motor perception as well. Visual feedback of participants’ finger movements was manipulated using virtual reality to be anatomically congruent or incongruent to the performed movement. Participants made a motor perception judgment (i.e. which finger did you move?) or a visual perceptual judgment (i.e. which finger did you see moving?). Subjective measures of agency and body ownership were also collected. Seeing movements that were visually incongruent to the performed movement resulted in a lower accuracy for motor perception judgments, but not visual perceptual judgments. This effect was modified by rotating the virtual hand (Exp.2), but not by passively induced movements (Exp.3). Hence, sensorimotor conflicts can modulate the perception of one’s motor actions, causing viewed “alien actions” to be felt as one’s own.

Our sensation of control over our actions and movements, termed agency, is an important factor in self-consciousness^1^, allowing to distinguish oneself from the environment and from other people^2^,^3^,^4^,^5^,^6^. This sense of agency is thought to be based on the integration of efferent (motor) and afferent sensory (e.g. visual, proprioceptive, tactile) signals^6^,^7^,^8^. Current theoretical models suggest that agency arises from the correspondence of the predicted with the actual sensory outcomes of our movement^9^,^10^,^11^. Thus, when we make a movement it has been argued that the brain compares the actual sensory consequences with an internal model of the predicted sensory consequences of that movement^12^. When the predicted sensory consequences match the incoming sensory signals the movement is attributed to the self and a sense of agency over the movement is established^10^,^13^. Indeed, during sensorimotor conflicts, in which there is a mismatch between performed and observed actions, people typically feel a loss of agency, attributing the movement to an external source^4^,^9^,^14^.

However, there is evidence that the sense of agency may also be an inferential process whereby the feeling of control is inferred based on contextual information^15^,^16^,^17^,^18^. For example, one study showed that participants could feel agency over hand movements of another person when they heard verbal information instructing the movements of the ‘alien hands’ and when they were placed in an anatomically plausible position^17^. Other studies have shown that agency may be modulated by many factors including priming^15^,^16^,^19^, outcome value^18^ and prior beliefs^20^. These studies indicate that inferential and postdictive cues may have a strong effect on agency^21^, leading to alternative theories of agency based on optimal cue integration, in which agency cues are weighted by their reliability, and based on the specific context^22^,^23^.

The notion that the feeling of agency relies on the integration of efferent motor information with afferent sensory information, indicates the strong link between the processing of motor and perceptual signals. The common coding theory^24^,^25^, based upon the ideomotor principle, states that actions and perceptions of actions are coded in a common representational format. This theory predicts bi-directional influences between action and perception which can exert either an excitatory (e.g. action facilitates perception^26^,^27^,^28^) or an inhibitory effect (e.g. action impairs perception; cf.^29^). Thus, action and perception have reciprocal effects, thought to be driven by the use of common neural systems^27^,^30^,^31^,^32^. This common representation of perception and action could pose a problem for discriminating between one’s own actions and perceived actions of others (i.e. as when perceiving someone else’s action or when performing a joint action; cf.^33^). It has been suggested that this is resolved within the sensorimotor system by using prediction signals associated with self-generated movements (i.e. efferent motor signals and afferent bodily signals, e.g. proprioception, tactile information^34^). Indeed when perceived movements deviate too strongly from one’s intended or predicted movement, the feeling of agency is reduced and the movement is attributed to an external source^2^,^3^,^5^ . However, other cues such as visual aspects or the posture of the limb^17^,^35^,^36^,^37^ related to the sense of ownership may also help to discriminate between one’s own and another’s actions. Indeed, previous studies using the rubber hand illusion have shown that congruent visuo-tactile stimulation may cause visual dominance over proprioceptive information leading to a proprioceptive mislocalization based on the visual information^36^,^38^.

These findings have recently been framed in a predictive processing framework of self-recognition, according to which the brain continuously makes predictions and inferences about sensory input from the body and the environment, to infer the most likely model of the self^39^. More specifically, higher-level multimodal brain areas aim to minimize ‘prediction error’ signals from lower-level sensory areas in a Bayes-optimal fashion, through the continuous updating of generative models of the ‘self’. Hierarchically organized models are in turn used to make predictions regarding both exteroceptive and interoceptive signals and feed-forward prediction-error signals are used to update these models^40^,^41^. Thus, according to the predictive processing framework, prior experiences of the body and the environment are at the basis of a bodily self-model that generates predictions about what the body and the world will be like in the future. Importantly, the predictive processing model also allows to account for the above-mentioned findings that agency can be inferred based on inferential and post-dictive cues: in these cases novel information (e.g. incoming verbal input) is used to update one’s model about one’s body and the actions that one controls.

Given the abovementioned strong link between perception and movement and the importance of prediction error signaling for generating a ‘self-model’ and for feelings of agency, it could be that a mismatch between a movement and the resulting sensory information may result in a change in the perception of one’s motor or sensory representations as well. Following the notion that the brain constructs a coherent model of the self through the integration of multisensory information^39^, it could well be that visual sensory information during a sensorimotor conflict modifies motor perception, causing us to incorporate ‘alien actions’ into our motor representation, thereby selective overruling efferent and afferent non-visual bodily signals. Such a finding would provide an important new contribution to our understanding of sensorimotor mechanisms underlying the experience of agency, indicating that sensorimotor conflicts do not only affect feelings of agency (i.e. ‘was I the one who was making the movement?’) but also one’s motor perception (i.e. ‘which finger did I move?’). Indeed, while several studies have reported the modulation of the sense of agency through sensorimotor conflicts^8^,^37^,^42^,^43^,^44^,^45^, indicating the boundary conditions under which actions are ascribed to the self, there has been little research on how such sensorimotor conflicts may affect perception and memory for visual and motor signals involved in these actions. For example it is possible that a sensorimotor conflict may modulate visual perception to match the motor action (motor capture of vision), or conversely that the perception of the motor action may be modulated by the visual information (visual capture of action). Critically, this can only be tested using a paradigm concurrently measuring both effects within the same design such as to not bias the weighting of the motor and sensory cues as would occur if only visual or motor perception was tested^46^. Thus, the aim of the present study was to investigate the effects of sensorimotor conflicts on motor and visual perception and how such effects relate to the feeling that the movement is related to the self.

Here we employed a novel paradigm allowing the testing of concurrent bidirectional influences of sensorimotor conflicts on visual and motor perception of finger movements. We employed an event related design in which on each trial the participants moved their index or middle finger. A three dimensional virtual hand was superimposed over the participant’s real hand location. On each trial the virtual hand was programmed to make either the same finger movement (*congruent*-e.g. index-index) or an incongruent movement (e.g. index-middle). For each trial participants were requested to report either which finger they had moved (*Motor Perception task*) or which finger they had seen moving (*Visual Perception task*). Subjective agency and body ownership ratings were also collected, as this allowed us to relate changes in the perception of seen or performed movements to the subjective feeling that the seen hand was one’s own. We used this paradigm in three experiments to investigate the basic effect of sensorimotor conflicts on visual and motor perception (Experiment 1). We then explored the effect of spatial congruency of the seen and actual hand on *Motor Perception* by rotating the orientation of the virtual hand (Experiment 2). Finally, we tested the role of efferent information on *Motor Perception* by comparing active vs. passive finger movements (Experiment 3). We predicted that incongruent visual-motor feedback would affect the retrospective *Motor Perception* but not judgment of visual perceptions and that this would be modified by ownership of the virtual hand. For exploratory purposes we also analyzed reaction times, as previous studies have shown that sensorimotor conflicts can also affect the speed of responding^47^. We further hypothesized that incongruent feedback would modulate both the sense of ownership over the hand and the sense of agency over the movements.

## Methods and Materials

### Participants

Participants were 55 healthy volunteers (Exp. 1: N = 20, 9 males, mean age: 23.7 ± 3.5 years; Exp.2: N = 20, 16 males, mean age: 23.7 ± 3.7 years; Exp.3 : N = 15, 10 males; mean age: 24.5 ± 4.5)) from the student population at Ecole Polytechnique Fédérale de Lausanne (EPFL) and at the University of Lausanne in Switzerland (UNIL). All participants had normal or corrected-to-normal vision, were right-handed, had no psychiatric or neurological history and were naïve with respect to the purpose of the study. They participated in the study for payment (30 CHF, about 30 USD). All participants gave informed consent and the study was approved by the ethics committee of EPFL. The study and methods were carried out in accordance with the guidelines of the EPFL ethics committee and the guidelines of the declaration of Helsinki.

### Materials

#### Virtual Hand

**Figure 1:**
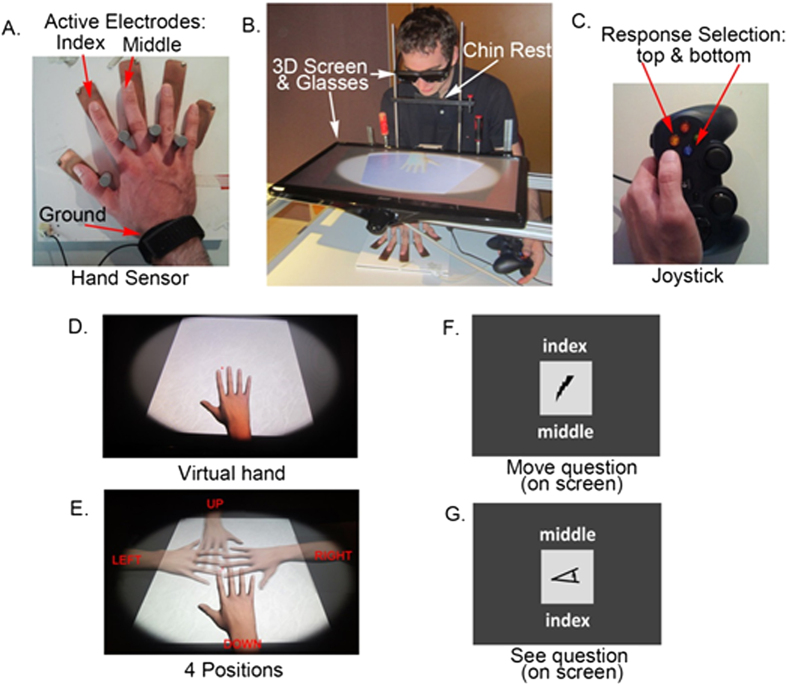
Experimental setup. (**A**) The hand sensor used for recording finger movements. (**B**) The whole setup with the 3D screen and glasses and the chin rest. (**C**) The joystick used to answer the questions. (**D**) The virtual hand as it appeared on the screen for experiments 1 & 3 and the 0° position in experiment 2. (**E**) An illustration of all four positions (0°, 90°, 180° and 270°), which were tested during the second experiment. Note, only one hand orientation was shown in each condition. (**F**,**G**) The icons cueing the *Motor Perception* task (**F**) corresponds to the question “Which finger did you move?”. The icon cueing the *Visual Perception* task (**G**) corresponding to the question “Which finger did you see moving?”.

The three dimensional animation models of the virtual right hand (16 × 11 cm as it appeared on the screen, from thumb to pinky and from the wrist to the top of the middle finger, respectively) were custom built with 3D modeling software (Autodesk 3D Studio Max, SmithMicro’s Poser, and the open source program Blender). The virtual hand as it appears on the screen (DOWN positions) is represented in Fig. 1D. The hand was seen in 3D using a 3D screen with 3D glasses as described below. To customize the animation of movement of the virtual hand, the angle and speed of finger movement were adjusted manually.

#### Hand sensor

To track movement onsets, the virtual hand was coupled to a custom-made sensor consisting of a wooden tray (25 × 25 cm) with five electrodes made of copper (width: 2.5 cm and length: 6, 8, 9, 9 and 6 cm, going from thumb to pinky) connected to a wristband with a 2.5 × 2 cm ground electrode. The sensor can be seen in Fig. 1A. The sensor was connected with an Ethernet cable to an Arduino Uno microprocessor, which was connected to the computer by a USB cable. By measuring the electrical conductivity on each electrode this device informs in real-time which of the five fingers is in contact with the copper strip. To avoid lateral hand movement during the experiments, vertical cylinders were installed between the electrodes, in order to ensure normal finger spacing and a natural hand position. The hand sensor was positioned about 10 cm away from the table edge in front of the subject.

#### Display

Stimuli were displayed on a 3D screen (Acer HN274, 27 inches) and presented using custom made software (ExpyVR; http://lnco.epfl.ch/expyvr) and viewed via 3D glasses (NVIDIA 3D vision 2, model P1431). The software ran on a computer with 64-bit Windows 7 (Intel(R) Core(TM) i7 CPU 860 @2.80). The 3D screen was positioned above the subject’s hand at a distance of 17 cm above the table. A chin rest, whose height could be manually adjusted between 20 and 25 cm, was installed in front of the screen. The full setup can be seen in Fig. 1.B.

#### Response device

Participants’ responses were collected using two buttons of a commercial gaming joystick (Logitech Gamepad F310) that the participant held in the left hand, as can be seen in Fig. 1C.

#### Questionnaire

A questionnaire, adapted from previous studies on body ownership and agency e.g.^7^, was administered to the participants as described in the Procedure section. First some demographic information was collected. Then questionnaires with items measuring agency judgments and ownership judgments were administered. Agency questions included the following items: (*I felt as if I was causing the movement I saw. The* virtual hand *moved just like I wanted it to, as if it was obeying my will. I felt as if I was controlling the movements of the* virtual hand*. Whenever I moved my finger I expected the virtual finger to move in the same way*.). Ownership questions included: (*I felt as if I was looking at my own hand. It seemed as if I were sensing the movement of my finger in the location where the virtual finger moved. I felt as if the* virtual hand *was part of my body. I felt as if the* virtual hand *was my hand*.). The same questions were administered for congruent movements and incongruent movements. Subjects had to answer on a seven-point Likert scale ranging from 1 = Totally disagree/7 = Totally agree.

### Procedure

When the participant arrived in the experimental room, he was shown the room with the setup and the task was briefly explained. Then the participant signed a consent form. Next, the participant was positioned in front of the screen with the 3D glasses on and with his hand on the hand sensor with the wristband attached to his arm. He was then asked to freely move either his index or middle finger while looking at the virtual hand. The participant was asked to fixate on the red dot between both fingers (see Fig. 1D) and move his fingers. This practice session, in which the virtual hand always displayed the congruent movements as performed by the participant, lasted 2 minutes.

Then the participant was introduced to the experiment and started by training on a block of 48 trials. For each trial the subject could freely lift and put back down either his middle or index finger. The virtual hand simultaneously lifted either the same finger (*congruent* condition) or the other finger (*incongruent* condition). Immediately after the virtual hand’s lifting and repositioning of the finger, a question appeared on the screen. On each trial one of two possible tasks were randomly selected: a *Visual Perception task* or a *Motor Perception task*. In the *Visual Perception task* participants had to indicate which finger they saw moving (cued by a white square with a black eye drawn on it. Fig. 1G). In the *Motor Perception task* participants had to indicate which finger they had moved (represented by a white square with a black thunderbolt drawn, Fig. 1F). The two possible answers (index or middle) appeared each on top and on the bottom of the white square. To control for stimulus–response compatibility the software also randomly chose the position of the answers (index on top, middle on the bottom or the inverse). Thus, the participants didn’t know in advance if they were required to report the motor or visual aspect of the movement. Responses were given by pressing one of two buttons on a joystick with the left thumb (Fig. 1C). The reaction times (RT) were measured between the time at which the question appeared up to the moment when the participant pressed a button on the joystick.

After the training session, the participants began the main experiment, which consisted of 320 trials divided into 3 blocks. Participants were asked to answer as quickly and accurately as possible. They were also instructed to balance as much as possible the number of index and middle finger movements. To assist with equating the index and middle finger movements, a screen appeared at the middle of each block (after 60 trials), which indicated how many times they moved each finger. After each block, the participants had to answer the agency and ownership questions, once for the *congruent* trials and again for the *incongruent* trials. After the experiment, the participants were thanked, given their remuneration and debriefed.

### Experiment 2

The setup was identical to that of experiment 1 except that we used four versions of the virtual hand orientation. The three dimensional animation models of the virtual right hand (16 × 11 cm as it appeared on the screen, from thumb to pinky and from the wrist to the top of the middle finger, respectively) were rotated by 0, 90, 180 and 270 degrees while keeping the spatial distance from the central fixation spot constant. The experiment was divided in different blocks, with different virtual hand orientations: the hand was either rotated at 0° (Down position), 90° (Right position), 180° (Up position) or 270° (Left position) counterclockwise. Figure 1E. illustrates at once the four possible virtual hand positions, which appeared alternatively in different blocks. The participants conducted 8 blocks of finger lifting and question answering. In each block, there were 80 trials, and the position of the virtual hand was kept constant within each block. Each of the four positions of the virtual hand (0°, 90°, 180° and 270°) was tested twice. The order of the rotation angle for each block was randomized. After each block, the participants had to answer the general questionnaire. This questionnaire is made of 2 sets of 4 questions, one set for the congruent items and one set for the incongruent ones. In each set of 4 questions, 2 questions were items about agency, and 2 about ownership.

### Experiment 3

The setup was identical to that of experiment 1 except for the addition of a passive condition in which the participant’s finger was moved passively by the experimenter. The experiment was divided in 4 different blocks, 2 blocks of active finger lifting and 2 blocks of passive finger movements. The active condition was identical to that of experiment 1. In the passive condition a trained experimenter used a small metallic lever to elevate the finger of the participant. Special care was taken to create similar movement dynamics between the active and passive conditions and the number of index and middle finger movements was kept balanced by feedback between blocks as in experiments 1 & 2. All other parameters were identical to those of experiment 1. In each block, there were 80 trials. The order of the conditions was randomized. After each block, the participants had to answer 2 sets of 4 questions, one set for the *congruent* items and one set for the *incongruent* ones. In each set of 4 questions, 2 questions were items about agency, and 2 about ownership.

### Statistical analysis

For all trials, the accuracy and response times (RT) were measured. For the analysis RTs were kept only for correct answers and they were discarded if they were 2.5 standard deviations away from the subject’s mean RT (percentage of trials discarded: 2.7% of all trials). Accuracy and RTs were analyzed with a 2 × 2 repeated measures ANOVA with the congruency of the virtual hand movements (*congruent*/*incongruent*) and the type of question (*Visual Perception*/*Motor Perception*) as within subject factors. Post-hoc tests were done using fisher’s LSD when required. In the questionnaires, responses for the agency and ownership items were averaged separately to allow a single composite measure of these domains. The questionnaire ratings were tested for normality using the Kolmogorov-Smirnov & Lilliefors test and then analyzed statistically with a paired t-test or Wilcoxon matched-pairs test as required. Significant effects were reported for *p* < 0.05. In experiment 2, RT and accuracy rates were subjected to a 4 (rotation: 0°/90°/180°/270°) X 2 (Displayed Movement: *Congruent*/*Incongruent*) X 2 (task: *Visual Perception*/*Motor Perception*) repeated measures ANOVA. Questionnaire scores were analyzed as in exp. 1. In experiment 3, RT and accuracy rates were subjected to a 2 (movement type: Active/Passive) X **2** (Displayed movement congruency: *Congruent*/*Incongruent*) X **2** (task: *Visual Perception*/*Motor Perception*) repeated measures ANOVA. Questionnaire scores were analyzed as in exp. 1. All reaction time results can be viewed in the supplemental material.

## Results

### Experiment 1

#### Accuracy

**Figure 2:**
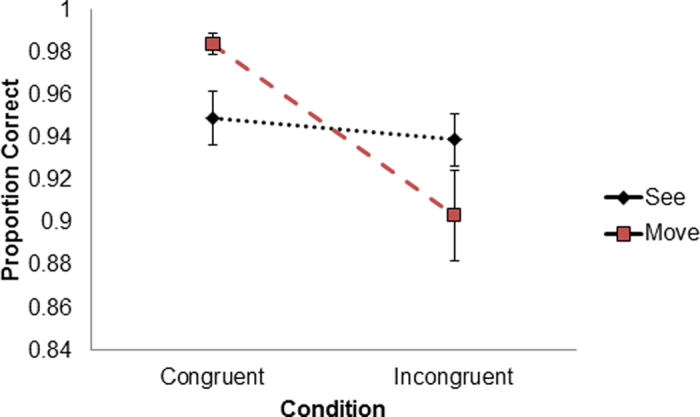
Accuracy by congruency and task. Interaction between the task (*Visual Perception*/*Motor Perception*) and the anatomical congruency (*congruent*/*incongruent*) between the visual feedback and the movement performed. Note for the *Visual Perception* task no difference was found for *congruent* and *incongruent* information, while the *Motor Perception* task accuracy in the *incongruent* condition was significantly reduced. Error bars denote SEs.

Overall accuracy in the experiment was high (*M* = *94.3%, SE* = *0.01*) indicating that the participants were able to perform the task despite its complexity. Statistical analysis revealed a main effect for anatomical congruency (*F*(*1,19*) = *13.75*, *p* < *0.001*, *η*^2^ = 0.41). Participants were more accurate for trials in which the virtual hand and real hand finger movements were *congruent* (*M* = *96.6%, SE* = *0.9%*) than in the *incongruent* situation, when the visual feedback was different from the finger they moved (*M* = *92.1%, SE* = *1.7%*). No main effect was found for the task type (*Visual Perception*/*Motor Perception*) (*F* < *1*) with similar accuracy rates for *Visual Perception* (*M* = *94.3%, SE* = *1.3%*) and *Motor Perception* conditions (*M* = *94.3%, SE* = *1.3%*). Critically, the analysis revealed a significant interaction between the task (*Visual Perception*/*Motor Perception*) and the anatomical congruency (*congruent*/*incongruent*) (*F*(*1,19*) = *11.98, p* = *0.003, η*^2^ = 0.38) (Fig. 2).

Post hoc analysis indicated that the interaction was driven by differences between *congruent Motor Perception* trials and *incongruent Motor Perception* trials. Trials in which participants were required to report which finger they moved (*Motor Perception* task) were more accurate when the real movement and virtual hand movement were anatomically *congruent* (*M* = *98.4, SE* = *0.4%*), compared to *incongruent* trials, where participants made more errors in reporting which finger they moved (*M*  = *90.3%, SE* = *2.1%, p* < *0.0001*). No significant difference was observed between *congruent Visual Perception* trials (*M* = *94.8%, SE* = *1.2%*) and *incongruent Visual Perception* trials (*M* = *93.8%, SE* = *1.2%, p* > *0.4*).

#### Questionnaire results

**Figure 3:**
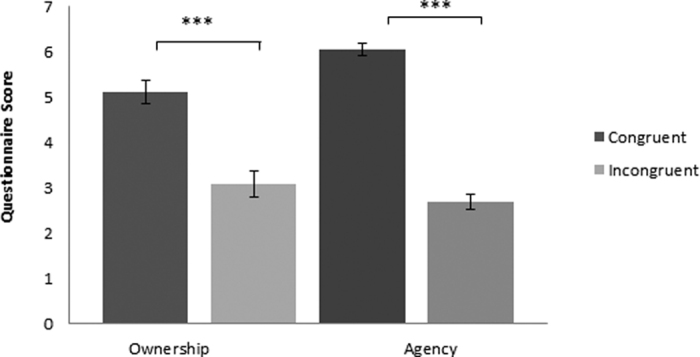
Questionnaire score for Ownership and Agency. Mean subjective ratings of ownership and agency (range: 1 = not at all; 7 = very much) for *congruent* and *incongruent* conditions. Errorbars denote SE. ***p < 0.001.

The questionnaire results revealed a significant difference for *Ownership* between the *congruent* (*M* = *5.11, SE* = *0.26*) and *incongruent* conditions (*M* = *3.08, SE* = *0.29, p* < *0.0001*). As expected, ratings for *Agency* also showed a significant difference (*p* < *0.0001*) with higher ratings in the *congruent* (*M* = *6.05, SE* = *0.14*) than for the *incongruent* (*M* = *2.67, SE* = *0.17*) trials (see Fig. 3).

### Discussion

The results of Experiment 1 showed that visual perceptual judgments were not affected by the congruence between performed and observed actions, as demonstrated by the flat slope in Fig. 2. In contrast, motor perception judgments were significantly affected by the congruence between performed and observed movements. When participants saw an incongruent digit moving (with respect to the digit they actually moved) this resulted in more errors in reporting which finger they had moved. This finding indicates that visual information may selectively overrule action information (based on motor, proprioceptive and tactile signals) thereby resulting in more incorrect judgments regarding which finger was moved.

In this first experiment, the congruency of actions was always defined in terms of the spatial compatibility between the performed and the seen movement. Accordingly, an important question is to what extent the effects of congruency on action judgments are primarily related to effects of interdigit spatial compatibility, driven by the bidirectional learned associations between actions and their visual consequences (i.e. moving the index finger is usually associated with observing an index finger movement). That is, many studies have shown automatic effects of spatial information on action planning^26^ and on action judgments^2^,^3^,^48^,^49^. On this account, the interference of observed actions on action judgments may reflect an automatic effect of learned spatial response-effect associations: for example observing a movement of the index finger activates the response code for index finger movements and results in confusion regarding which finger was moved^24^. Alternatively, the ownership ratings in the first experiment provide tentative support for the notion that ownership of the hand underlies the effect. That is, participants reported stronger feelings of ownership for the hand for congruent compared to incongruent movements. It could be that the interference effect of seen finger actions on finger judgments is related to the varying levels of ownership of the virtual hand. Therefore, in order to obtain more direct evidence for the importance of ownership of the virtual hand for the observed effects, in the second experiment we manipulated the orientation of the virtual hand, which has been shown to affect the sense of ownership^5^.

In the second experiment we presented participants with virtual hands in different orientations (i.e. up, down, left, right) (see Fig. 1A), while keeping their real hand position constant. In this way, the position of the virtual hand could be anatomically compatible or rotated with the participants’ real hand, while at the same time we also manipulated the spatial mapping between the real and the virtual hand. If the effects in the first experiment are primarily related to the spatial overlap between the real and virtual hands we should expect no effect of observed movements on motor perception for hands rotated 90° and 270° inverted hands which are both inverted by 90° compared to the real hand. In contrast, if the effects are driven more strongly by the ownership of the virtual hand, we should expect interference effects only for positions for which there is ownership for the virtual hand (the 0° and the 90° position). To this end, in experiment 2 we modulated the sense of ownership over the virtual hand by rotating it by 0, 90, 180 and 270 degrees from the orientation of the real hand^5^. Results from mental rotation studies show that hands rotated in a medial direction (90 degrees counterclockwise for right hands) are anatomically plausible and can therefore be easily embodied and mentally rotated e.g.^50^, whereas mental rotations of hands in a lateral direction (270° counterclockwise for right hands) are more difficult to carry out. Moreover, previous studies suggested that ownership has a strong impact on action to perception or perception to action processing^5^,^28^,^51^. In this way we could assess the impact of the sense of ownership over the virtual hand on motor and perceptual judgements. Furthermore, by presenting hands in a spatially compatible (0° and 90°) and incompatible (180° and 270°) position, the effects of spatial compatibility on motor and perceptual judgments could be directly investigated.

### Experiment 2

#### Results

##### Accuracy

**Figure 4:**
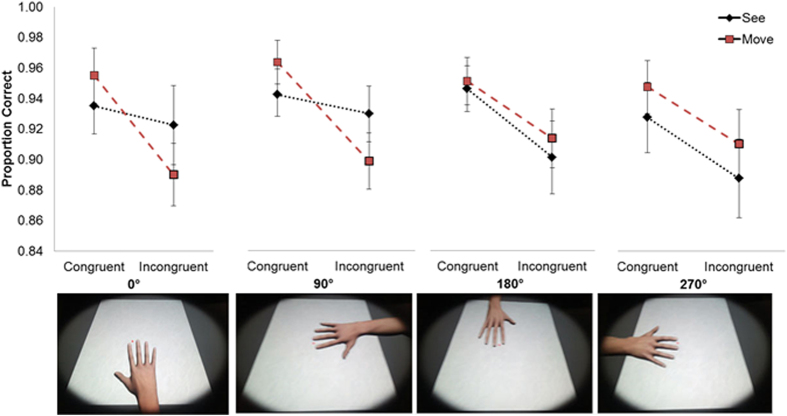
Accuracy for the *Visual Perception* and *Motor Perception* tasks for all rotations as a function of congruency. Interaction between the task (*Visual Perception*/*Motor Perception*), the anatomical congruency between the visual feedback and the action performed (*congruent*/*incongruent*) and rotation (0°, 90°, 180° and 270°). Note that for the anatomically possible rotations (0°, 90°) the results replicate those of experiment 1. However in the unnatural rotations (180°, 270°) a different action-perception dynamic is revealed. Errorbars denote SEs.

The 4 × 2 × 2 three-way repeated measurement ANOVA revealed a main effect of Congruency (*F*(*3, 57*) = *26.76, p* < *0.001, η*^*2*^ = 0.59), reflected in higher accuracy rates for *congruent* trials (*M* = *0.94, SD* = *0.1*) compared to *incongruent* trials (*M* = *0.90, SD* = *0.1*). Additionally, an interaction between the task type (*Visual Perception* or *Motor Perception*), Congruency (*congruent* vs. *incongruent* trials) and rotation (0°, 90°, 180° and 270°) was found (*F*(*3, 57*) = *5.07, p* < *0.005, η*^*2*^ = 0.21). As can be seen in Fig. 4, the 3-way interaction is reflected in a differential effect of congruency on response accuracy for the *Visual Perception* task, between the different experimental conditions. In the 180° and 270° rotations response accuracy was significantly modulated by visuo-motor congruency with lower accuracy in the incongruent condition (180°: M = 0.90, SD = 0.08; 270°: M = 0.88, SD = 0.1) than in the congruent condition (180°: M = 0.94, SD = 0.1, p < 0.0001; 270°: M = 0.88, SD = 0.1, p < 0.001). No such difference was found for *Visual Perception* task in the 0° and 90° rotations (*p* = *0.24 & p* = *0.23* respectively), as also found for the 0° in experiment 1. The accuracy in *Motor Perception* task however was influenced by visuo-motor congruency in all rotations (0°: p < 0.00001, 90°: p < 0.0001, 180°: p < 0.001, 270°: p < 0.001). All other effects were not significant (*F* < *2.14*).

To further explore the 3 way interaction, four separate 2 (*Visual Perception*/*Motor Perception*) X 2 (*Congruent*/*Incongruent*) repeated measures ANOVAs were performed for each position of the virtual hand. For the 0° and 90° conditions a significant interaction between the task type and congruency was found (0°: (*F*(*1,19*) = *9.22, p* < *0.05*; 90°: *F*(*1,19*) = *6.50, p* < *0.05*). However, this interaction was not significant in the 270°- and the 180°-orientations (270°: *F*(*1,19*) = *0.26, p* = *0.61*; 180°: *F*(*1,19*) = *0.01, p* = *0.91*). The interaction in the 0° and 90° rotation was driven by a large difference in the accuracy in the *Motor Perception* task between the congruent and incongruent conditions (p < 0.0001 and p < 0.001 respectively) while the *Visual Perception* task was not modulated by congruency (p = 0.31 and p = 0.4 respectively). In the 180° and 270° rotations only the main effect of *Congruency* was significant (p < 0.01 and p < 0.01 respectively) with lower accuracy in the incongruent conditions. Based on these two different response profiles for the anatomically possible rotations (0° and 90°) vs. anatomically impossible rotations (180° and 270°) we then compared the effects of visuo-motor congruency and task type between them. Post-hoc t-tests confirmed that in the 0° and 90° conditions for the *Visual Perception* task there was a smaller difference between *congruent* and *incongruent* trials (*M* = *0.01, SD* = *0.03*) compared to the 180°- and 270°-rotation conditions (*M* = *0.04, SD* = *0.05, p* < *0.01*). Conversely, the slopes of the *Motor Perception* task (*Motor Perception congruent*-*Motor Perception incongruent*) were shallower for the 180° and 270° conditions (*M* = *0.03, SD* = *0.05*) than for the 0° and 90° conditions (*M* = *0.06, SD* = *0.02, p* < *0.05*).

##### Questionnaire

**Figure 5:**
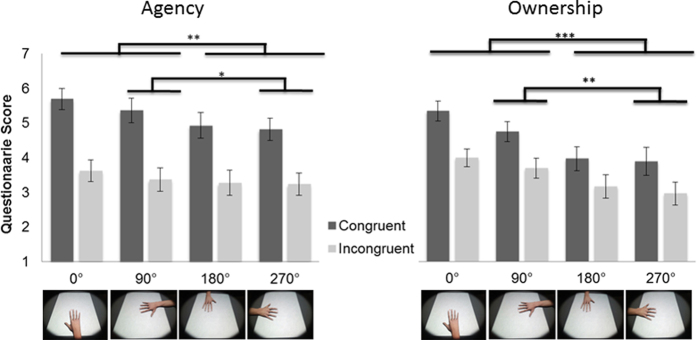
Mean questionnaire scores for agency (left) ownership (right) and questions, for each hand position (0°, 90°, 180° and 270°) for *Congruent* and *Incongruent* trials. Note significant decrease in ownership and agency ratings for unnatural hand positions (Up & Left) as well as for *Incongruent* trials. Errorbars denote SEs.

Questionnaire scores revealed that for both *congruent* and *incongruent* trials, ownership scores were significantly higher (*z* = *3.86, p* < *0.001*) in the natural virtual hand positions, 0° and 90° (*M* = *4.6, SD* = *1.1*), compared to the two positions that are physically difficult to reach by physical rotation of the hand (180° and 270°) virtual hand positions (*M* = *3.6, SD* = *1.4*). Because the 0° position matches the physical hand’s position, the 270° position was also directly compared to the 90° position. It was found that the ownership feeling was higher in the 90° position (*M* = *4.4, SD* = *1.2*) than in the 270° position (*M* = *3.5, SD* = *1.5*), although both orientation conditions differ by the same orientation angle from the 0° condition (Fig. 5).

Similar tests were performed for agency questions, and showed that the 0° and 90° positions had higher agency scores (*M* = *4.5, SD* = *1.2*) than the physically impossible 180° and 270° positions (*M* = *4.1, SD* = *1.3; z* = *2.98, p* < *0.01*). Similarly, for the 90° position a higher sense of agency (*M* = *4.3, SD* = *1.3*) than for the 270° position (*M* = *4.0, SD* = *1.2*) was observed (*z* = *2.33, p* < *0.05*). Importantly, to test if physically impossible or unnatural hand positions affect ownership more than agency we tested the difference in ownership and agency scores between the natural virtual hand positions (0°, 90°) and the anatomically impossible positions (180°, 270°). The results indicated that ownership was more strongly modified by the orientation of the virtual hand than agency (*z* = *2.07, p* < *0.05*). These data confirm our hypothesis that the orientation of the hand modulates the sense of ownership over the virtual hand. Particularly the 270° and 180° positions create reduced feelings of ownership compared to the 0°and 90° positions. Correlations between changes in subjective ownership and agency and *Visual and Motor Perception* tasks did not reach significance (See supplementary material for details).

### Discussion

In the second experiment we investigated whether the effects of observed actions on motor judgments were driven primarily by spatial compatibility or by ownership of the virtual hand. Our findings suggest that experimental conditions associated with changes in ownership for the virtual hand may underlie the effects of action observation on *Motor Perception* judgments. As expected, participants reported stronger feelings of ownership and agency when the virtual hands were presented in an anatomically plausible (0, 90°) compared to an implausible posture (180°, 270°).

Furthermore, we observed a differential interference of observed actions on action judgments in the anatomically plausible compared to the implausible conditions. Critically, when the virtual hand was placed in an anatomically possible position it was found that motor judgments were more strongly affected by the observed action than Visual Perception judgments. However, when the virtual hand was placed in an anatomically impossible position observed actions equally affected perceptual and motor judgments. These findings argue against the possible confound that spatial compatibility could underlie the effects observed in the first experiment, but suggest the importance of the identification with the virtual hand for effects of visual perception on *Motor Perception* to occur.

The results of Experiment 2 showed that the rotation of the virtual hand affected the sense of ownership over that hand. Interestingly, this manipulation of ownership affected action perception interactions. For the 0° and 90° rotations ownership scores were high and the action-perception interaction replicated the effects that were found in experiment 1. More specifically, it was found that the *Visual Perception* task was unaffected by which finger was moved as can be seen in the relatively flat slopes between the *congruent* and *incongruent* conditions (Fig. 4). The results for the *Motor Perception* task with these hand orientations also replicated the results of experiment 1, reflected in more errors about which finger was moved in the incongruent compared to the congruent conditions. However, in the non-plausible hand positions, where the ownership scores were significantly lower, the action-perception interaction for the *Visual Perception condition* was different. Here the *Visual Perception* task was also affected by the congruency of the movement. There was a substantial reduction in accuracy when there was a mismatch between the seen movement and that made by the participant as can be seen by the lack of the 2 way interaction between Congruency and Task in the 270° and 180° positions. These data show that action-perception interactions were modulated by those rotations of the virtual hand that also modulated body ownership over the virtual hand. Interestingly, for the *Motor Perception* task an opposite effect was observed in which the low ownership rotations caused a smaller reduction in accuracy between the *congruent* and *incongruent* conditions. Thus, when the action is perceived as not belonging to the self but as belonging to another person (i.e. as suggested by the agency and ownership ratings), this causes less interference with judgments of our own action.

However, as the manipulation of virtual hand rotation affected both ownership and agency judgments (Fig. 5), the changes in action perception interaction could be related to the change in either agency or ownership; therefore in a third experiment we aimed to disentangle these factors. Here we tested this by using a design similar to that of experiment 1 but by adding a passive condition in which the movements were induced by the experimenter. Given the importance of efferent action information for making agency judgments^2^,^3^,^28^,^48^,^49^, we predicted that passive movements would induce a reduction in the sense of agency but not the sense of ownership over the virtual hand. Furthermore, we predicted that manipulations of agency would not modify action-perception interactions such that the results for both active and passive movement should be similar.

### Experiment 3

#### Results

Using the same criterion as in previous experiments, one participant was removed from the analysis due to low accuracy scores (more than 2.5 STD from the group mean).

#### Accuracy

**Figure 6:**
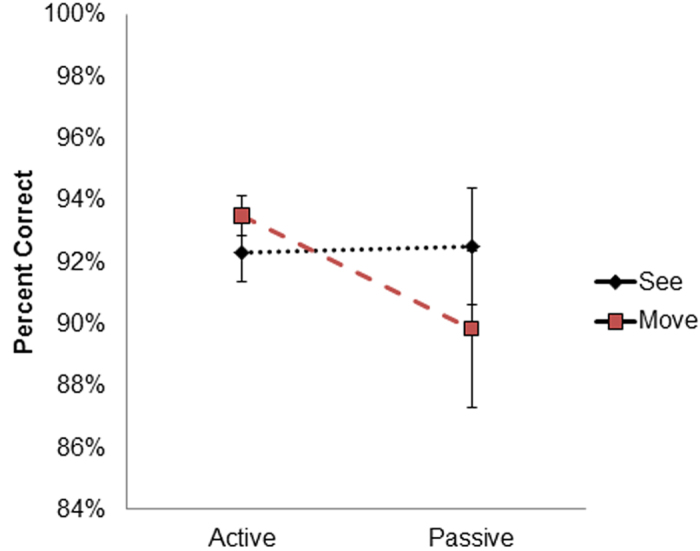
Accuracy by movement type and task. Interaction between the task (*Visual Perception*/*Motor Perception*) and the movement type (Active, Passive). Note for the perceptual task (*Visual Perception* condition) no difference was found for Active and Passive movements, while the action task (*Motor Perception* condition) accuracy in the Passive condition was significantly reduced. Errorbars denote SEs.

Accuracy analysis revealed a main effect of Congruency (*F*(*1,13*) = *16.54, p* < *0.01, η*^*2*^ = *0.56*): accuracy was higher for *congruent* trials (M = *0.95, SD* = *0.02*) than for *incongruent* trials (*M* = *0.88, SD* = *0.07*). As in experiments 1 & 2 the interaction between congruency and task was also significant (*F*(*1,13*) = *8.58, p* < *0.05, η*^*2*^ = *0.39*). The interaction was driven by a larger reduction in accuracy in the *Motor Perception* task between *congruent* (*M* = *0.96, SD* = *0.02*) and *incongruent* trials (*M* = *0.86, SD* = *0.07*) than in the *Visual Perception* task (*Congruent: M* = *0.94, SD* = *0.04, Incongruent: M* = *0.9, SD* = 0.08; *p* < *0.01*). The interaction between task and movement type was also significant (*F*(*1,13*) = *5.78 , p* < *0.05, η*^*2*^ = *0.30*. Participants showed a reduced accuracy for the *Motor Perception* task during passive movements (*M* = *0.89, SD* = *0.12*) compared to active movements (*M* = *0.93, SD* = *0.06*) with no modulation for *Visual Perception* judgments between the active (M = *0.92, SD* = *0.07*) and passive (*M* = *0.92, SD* = *0.06, p* < *0.05*, Fig. 6) conditions. The interaction between movement type and congruency was also significant ((*F*(*1,13*) = *5.29, p* < *0.05, η*^*2*^ = *0.28*). This interaction was driven by higher accuracy for active movements (*M* = *0.90, SD* = *0.07*) than for passive movements (*M* = *0.86, SD* = *0.11*) in the *incongruent* trials than between active movements (*M* = *0.95, SD* = *0.04*) than for passive movements (*M* = *0.96, SD* = *0.03*) in the *Congruent* trials (*p* < *0.05*). The three way interaction between Congruency, task and movement type was not significant ((*F*(*1,13*) = *1.78, p* = *0.2*) indicating that the interaction between task and movement type was not further modulated as a function of whether the actions were actively or passively performed. All other effects were not significant (*F* < *1.1*).

#### Questionnaire

**Figure 7:**
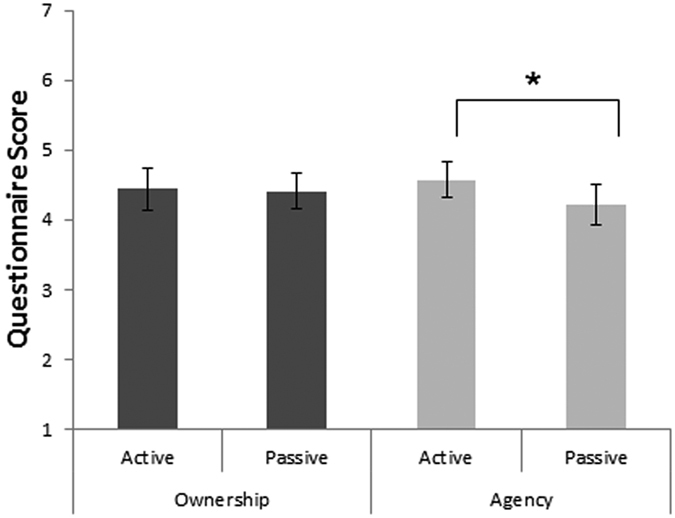
Mean questionnaire scores for ownership and agency questions, for active and passive movements. Bars denote standard errors. Data are collapsed across *congruent* and *incongruent* trials. *p < 0.05.

Questionnaire scores revealed an effect of inter-digit congruency on both agency and ownership ratings with higher ratings in the *congruent* (*M* = *5.29, SD* = *1.04*) than in the *incongruent* situations (*M* = *3.52, SD* = *1.33, t*(*13*) = *9.6, p* < *0.0001*, Fig. 7). Agency scores were higher (t(*13*) = *2.06, p* < *0.05*) in the Active condition (*M* = *4.57, SD* = *0.92*) than in the passive condition (*M* = *4.21, SD* = *1.05*). Ownership scores did not show any difference between the Active and Passive conditions (*p* = *0.42*).

### Discussion

The results of experiment 3 confirmed that when actions were not self-initiated but passively induced by another person this caused a modulation of the sense of agency as revealed by the questionnaire scores, but not in the sense of ownership over the virtual hand. For both active and passive movements in experiment 3, action-perception effects were similar to those observed in experiment 1 and in the high ownership conditions of experiment 2. For passive movements, there was a significant decrease in accuracy with respect to the active movement condition for the *Motor Perception* task while the same comparison in *Visual Perception* judgments was unaffected. This shows that when the action is not self-generated our judgments are more reliant on visual information, probably due to the lack of efferent information related to the movement. Contrary to our prediction, there was no three-way interaction between task, congruency and movement type, suggesting that the manipulation of agency (i.e. the hand being actively or passively moved) does not change the action-perception interaction.

## General discussion

In the present study we used a novel paradigm employing virtual reality to investigate whether induced sensorimotor conflicts can affect the perception of one’s movements. In addition, we investigated the relation between feelings of agency, feelings of ownership and perception-action interactions. We report three main findings. First, we found that judgments regarding one’s movement (which finger did you move?) were strongly modulated by visual information, whereas visual judgments (e.g. which finger did you see moving?) were unaffected by the congruency between performed and observed movement. Second, we found that the effects of observed movements on the judgment of movement were most pronounced when the virtual hand was presented in anatomically plausible positions. These findings suggest that the action-perception interaction is related to the sense of ownership over the virtual hand, which occurred only in the physically-plausible rotations. Finally, we showed that the effect of visual information on the judgment of movement occurred both for active and passive movements, thereby suggesting that awareness of one’s movements as well as proprioceptive consequences of one’s bodily movements, as conveyed by motor, proprioceptive and tactile signals can be overruled by conflicting visual information.

### Concurrent action-perception interactions

We found that when asked to judge which movement they had performed (*Motor Perception* task), participants’ accuracy was strongly affected by the movement they had viewed. When they viewed incongruent movements this resulted in a substantial reduction in accuracy for judging which movement they performed. This finding is quite surprising considering that the participants were free to choose the movement they performed with no time restriction: efferent signals, as well as tactile and proprioceptive signals related to the initiation, execution, and reafferent feedback of the action was available to them. Previous studies have shown that movement execution can be facilitated by congruent movement observation^26^,^47^. However our results go beyond these findings by showing that viewing an incongruent movement significantly reduces participants’ accuracy in reporting which movement they made, showing that the sensory consequences of an action may overrule efferent motor information.

### Modulation of action perception interactions by body ownership

In the second experiment we examined if the modulation of the action-perception interaction is driven by spatial compatibility or rather by the sense of ownership over the virtual hand. We manipulated ownership by rotating the virtual hand^5^ creating two anatomical positions which were compatible with possible self-rotations of the hand (0° and 90°) and two positions which are anatomically implausible and therefore more less likely to be embodied (180° and 270°)^52^. Subjective responses of participants showed that this manipulation indeed induced a change in ownership for the virtual hand. Importantly, for both of the high ownership rotations we replicated the pattern of responses found in the first experiment with visual perceptual judgments unaffected by movement, while the movement perception judgments were strongly affected by the viewed motion. This effect was different in the low ownership rotations, where both visual and motor perceptions were modified by the sensorimotor conflict, thus indicating the importance of ownership for processing of sensorimotor conflicts. Current theories of action representation have stressed the importance of shared representations between action and perception^24^,^33^. This has been supported by the discovery of mirror neurons in non-human primates^32^ and cortical regions that respond to both action execution and observation in humans^30^,^31^. These shared representations have been suggested to be involved in many processes of human cognition including social cognition, empathy, action understanding and imitation^53^,^54^. However, in light of these shared representations, it is not clear how self and other actions are segregated^11^,^49^. One study has shown that the attribution of the hand to the self or another agent modified the neural excitability of the motor system^55^ suggesting that this distinction is already resolved within the motor system. The results of experiment 2 show that action-perception interactions are strongly modulated by the viewpoint from which the hand is seen. Viewing the hand from an anatomically plausible position elicited high ownership ratings and strongly modified the results in the *Visual and Motor Perception* tasks suggesting that body ownership based on visual rotation angles may be an important cue for self–other segregation^3^,^5^. Indeed the neurobiological system related to action execution and action observation (the mirror neuron system) has shown to be sensitive to the viewpoint of the action^56^. A previous study in non-human primates has described different classes of mirror neurons responding to different viewpoints of action observation. This study showed that mirror neurons had different tuning curves for a first person viewpoint (i.e. 0°) compared to side views (i.e. 270°) and frontal views^56^. An additional study has shown stronger cortico-spinal excitability for observed action of a hand placed in a natural (no rotation) vs. an inversed position^57^. This suggests a neural basis for action-perception interactions based upon different anatomical rotations or viewpoints. It is possible that seeing the hand in physically impossible conditions causes higher mirror neuron system activation compared to rotations that are physically plausible for the self. Thus the effect of the sensorimotor conflict on the visual perception task for the low ownership rotations may stem from automatic ‘mirroring’ of the movements by the motor system.

Importantly, these findings can also be framed in terms of a predictive processing model of the bodily self, which integrates perceptual information to form a coherent model of the ‘self’: observed hand positions that are congruent with one’s proprioceptive hand position are more readily integrated in the self model than incompatible positions^58^. The inferred model of the self (i.e. ‘this hand is mine’) in turn selectively overrules potentially conflicting information (e.g. seeing a finger moving that does not correspond to one’s own movements), resulting in an updating of the prior model of one’s actions. A similar process has been proposed to account for body illusions for instance, in which the self-model based on the visual representation of a rubber hand tries to ‘explain away’ prediction error signals from one’s actual hand position, resulting in a mislocalization of one’s hand^39^.

### The role of efferent information in agency

The results of the last experiment show that when agency but not ownership is manipulated (by inducing a passive movement in half of all trials), there is no change in the action perception interactions (as indicated by the absence of a 3-way interaction). The results showed, as expected, a decrease in accuracy for the *Motor Perception* task in the passive condition in line with previous studies showing the importance of efferent information for the perception of own movements^2^,^59^. However, both active and passive conditions showed reduced accuracy for motor perception during sensorimotor conflict, suggesting that the effects of observed actions on movement judgments is not entirely dependent on efferent information (Exp. 3). Differences in motor perception accuracy between the *Congruent* and *Incongruent* conditions were present for both active and passive movements. This indicates that the visual capture of observed actions does not only override motor perception based on efferent information (i.e. motor commands), but also the information from somatosensory and proprioceptive information – which was comparable between active and passive movements. This result fits well with the notion that motor perception is not a unitary process involving only efferent motor information, but a multisensory process in which information from different modalities is integrated.

Previous studies on sensorimotor conflict and agency have typically induced these conflicts by inclusion of temporal^2^ or spatial deviations^7^,^60^. It has been shown that efferent information contains highly precise temporal information allowing fine grained judgments regarding action authorship^59^. In the current paradigm, there were no temporal delays between movement and feedback but rather the end effector was congruent or incongruent with respect to the performed action (i.e. index finger - middle finger). This finding has important implications for theories of motor control and the experience of agency, indicating that a mismatch between predicted and observed actions, may result in a prediction error signal that not only drives the updating of one’s sensory predictions (e.g. Wolpert *et al*.), but that may affect the representation of the movement as well. Recent theories of agency have suggested models e.g.^22^ incorporating both a ‘comparator model’ approach to agency in which predicted outcomes are compared with sensory outcomes^12^,^61^ with models suggesting that agency is based on inferences from action intentions^62^ or external information^15^,^16^,^17^ and the present findings are compatible with such an integrated model.

## Conclusions

Taken together the results indicate that when participants performed a motor perception judgment (i.e. ‘which finger did you move?’) these judgments were strongly modulated by visual information reflected in a reduced accuracy following the observation of incongruent compared to congruent movements. This basic finding reflects effects of perception on action, indicating that the retrieval of movement information is a constructive process, in which incoming visual sensory information modulates and selectively overrules afferent (tactile and proprioceptive) and efferent (motor command) information. Previous studies on motor perception have mainly focused on short-term motor memory, investigating for instance which specific factors (e.g. location, posture, verbal labeling) affect the retention of a movement over a prolonged period of time^63^,^64^. In contrast, the present study directly investigates the immediate memory for perception of a movement that one just performed (i.e. which finger did I move?). Moreover, here we employed a realistic virtual reality paradigm, which has been shown to effectively manipulate feelings of agency and ownership^65^,^66^, allowing us to study movement perception in an ecologically valid way compared to many previous studies using abstract movement cues e.g.^26^,^47^. Finally, our paradigm allowed manipulation of both body ownership (the feeling that he hand belongs to oneself) and agency (the sensation of control over the movement) which are considered fundamental aspects of the bodily self^1^. Thus, the current findings show that motor experience is modulated by visual signals and their incorporation within our body representation.

## Additional Information

**How to cite this article**: Salomon, R. *et al*. Changing motor perception by sensorimotor conflicts and body ownership. *Sci. Rep*. **6**, 25847; doi: 10.1038/srep25847 (2016).

## Supplementary Material

Supplementary Information
