# Supplementary material for: Changing motor perception by sensorimotor conflicts and body ownership
Source: Sci Rep. 2016 May 26;6:25847. doi: 10.1038/srep25847 (PMC4881011; doi:10.1038/srep25847)
Supplement: Supplementary Information [file srep25847-s1.doc]

**Supplemental information:**

**Changing motor perception by sensorimotor conflicts and body ownership**

Salomon, R^1,2*^., Fernandez, N.B.^2^, van Elk, M.^4^, Vachicouras, N^1,2^., Sabatier, F.^2^, Tychinskaya, A. ^5^ Llobera, J.^1,2^, Blanke, O.^1,2,3^

**Supplemental results**

**Experiment 1: Reaction times**

Reaction time (RT) analysis revealed a main effect of the task type (*F(1,19) = 9.37, p < 0.01,* ). Participants were faster to indicate which finger they moved (*M = 1.27s, SE = 0.08s*) than which finger they saw moving (*M = 1.34s, SE = 0.08s*). The analysis also showed a main effect of anatomical congruency (*F(1,19) = 17.97, p < 0.0001,* ), in which participants responded faster in *congruent* trials (M = 1.18s, SE = 0.10s) than in *incongruent* trials (*M = 1.43s, SE = 0.067s*). No interaction was found between the conditions in RTs (*p > 0.05*), indicating that RTs were not differently modulated by the anatomical congruency for the two tasks.

**Experiment 2: Reaction times**

RTs analysis showed a main effect of Congruency (*F(1,19)=29.07, p<0.001, η^2=^0.61*), reflected in faster RTs to *congruent* trials (*M=1.28s, SD=0.41s*) compared to *incongruent* trials (*M=1.73s, SD=0.46s*). The two-way interaction between congruency and task was nearly significant (*F(1,19)=4.11, p=0.055, η^2=^0.18*): for *congruent trials* participants responded faster in the *Motor Perception* task (*M=1.25s, SD=0.37s*) than in the *Visual Perception* task (*M=1.30s, SD=0.41s*), whereas for *incongruent trials* participants responded faster in the *Visual* *Perception* task (*M=1.70s, SD=0.43s*) compared to the *Motor Perception task* (*M=1.74s, SD=0.45s*). No other effects were significant (*F<2.14* ).

**Experiment 2: Correlations**

We explored possible correlations between the sense of ownership and agency and the modulation of the *Visual Perception* and *Motor Perception* task accuracy by congruency and rotation. For each participant we first calculated the difference in accuracy between the congruent and incongruent conditions for each task and rotation. We than computed the change in agency and ownership between the congruent and incongruent conditions for each task and rotation. We then computed the correlation coefficient between the change in agency scores (congruent-incongruent) and the change in accuracy (congruent-incongruent) for each task in each rotation separately. A similar computation was done for the ownership scores. Only one correlation was found to be significant (Ownership and Visual perception for 270° rotation, r=0.51 p<0.05) however no correlations survived when correcting for multiple comparisons.

**Experiment 3: Reaction times**

RTs analysis showed a main effect of Congruency ((*F(1,13)=16.56, p<0.005, η^2=^0.61)*: RTs were lower for *congruent* trials (*M=1.14, SD=0.21*) compared to *incongruent* trials (*M=1.48, SD=0.32*). The main effect for movement type approached significance (*F(1,13)=164.6, p=0.051, η^2=^0.26)* with faster responses for Passive trials (*M= 1.24 SD=0.22*) than in Active trials (*M=1.37, SD=0.32*). The main effect of task was also significant (*F(1,13)=11.92, p<0.005, η^2=^0.47)* with faster responses in the *Motor Perception task* (*M=1.25, SD=0.25*) than in the *Visual Perception task* (*M=1.36, SD=0.25*). No other effect reached significance (all *F<2.7*).
